# Comparative Efficacy of Pioglitazone, Saroglitazar, and Silymarin on Biochemical and Histopathological Hepatic Outcomes in a Wistar Rat Model of Non-alcoholic Fatty Liver Disease and Their Correlation With Insulin Sensitivity

**DOI:** 10.7759/cureus.103631

**Published:** 2026-02-14

**Authors:** Ipshita Jain, Rajendra Nath, Madhu Kumar, Rishi Pal, Rakesh Dixit

**Affiliations:** 1 Pharmacology and Therapeutics, King George's Medical University, Lucknow, IND; 2 Pathology, King George's Medical University, Lucknow, IND

**Keywords:** high-fat diet, insulin resistance, nafld, ppar, saroglitazar

## Abstract

Background: Non-alcoholic fatty liver disease (NAFLD) is the most common chronic liver disorder worldwide and is strongly associated with metabolic syndrome. Comparative evaluation of commonly used pharmacological agents is limited. This study aimed to evaluate and compare the effects of pioglitazone (PPAR‑γ agonist), saroglitazar (dual PPAR‑α/γ agonist), and silymarin on biochemical, anthropometric, and histopathological outcomes in a high-fat diet (HFD) Wistar rat model of NAFLD, and to assess their impact on insulin sensitivity

Material and methods: Thirty adult male Wistar rats were randomized into five groups (n=6 each): normal control (standard diet), NAFLD control (HFD), and three treatment groups receiving pioglitazone (10 mg/kg), saroglitazar (4 mg/kg), or silymarin (400 mg/kg) orally for 28 days after seven weeks of HFD administration. Outcomes assessed included body weight (BW) and liver weight (LW), serum lipid profile, alanine aminotransferase (ALT), fasting glucose, insulin, Homeostasis Model Assessment of Insulin Resistance (HOMA‑IR), and histopathological scoring (steatosis, inflammation, fibrosis).

Results: Saroglitazar consistently provided the greatest benefits, showing significant reduction in serum cholesterol, triglycerides (TG), low-density lipoprotein cholesterol (LDL‑c), very low-density lipoprotein cholesterol (VLDL‑c), ALT, and HOMA‑IR, with marked restoration of high-density lipoprotein cholesterol (HDL‑c) and improved histology. Pioglitazone was effective in improving insulin sensitivity and steatosis, while silymarin exhibited hepatoprotective effects but with relatively modest improvements.

Interpretation and conclusions: Saroglitazar demonstrated the most comprehensive protective effects against NAFLD progression, surpassing pioglitazone and silymarin in biochemical, histological, and insulin resistance parameters. Its dual PPAR‑α/γ activity may offer a more effective therapeutic approach, supporting further translational evaluation in NAFLD management.

## Introduction

Non-alcoholic fatty liver disease (NAFLD) has emerged as the most common chronic liver disorder worldwide and is increasingly viewed as a substantial public health burden. Current estimates suggest that approximately one in every three adults is affected, accounting for over a billion individuals globally, with higher prevalence observed in Southeast Asian and American populations [1,2]. NAFLD is diagnosed when hepatic steatosis, confirmed by histology or imaging, occurs in the absence of significant alcohol intake or other secondary liver conditions [3,4].

Over the past decade, NAFLD has been recognized not only as a hepatic condition but also as a multisystem metabolic disorder closely associated with obesity, insulin resistance, and type 2 diabetes mellitus [5,6]. Multiple risk determinants contribute to disease onset, including high-calorie diets, sedentary behaviour, alterations in gut microbiota, as well as genetic and epigenetic predispositions [7,8]. Among the mechanistic drivers, insulin resistance, chronic inflammation, and oxidative stress are considered critical in progression from simple steatosis to steatohepatitis, advanced fibrosis, and cirrhosis [9,10].

In 2023, the terminology was updated to metabolic dysfunction-associated steatotic liver disease (MASLD) to highlight the centrality of metabolic dysfunction and to avoid the stigma of the word “non-alcoholic” [11]. Despite its growing burden, the therapeutic armamentarium for NAFLD remains limited. Pharmacological agents studied to date are largely evaluated as monotherapies and head-to-head comparisons are scarce [12,13]. Lifestyle interventions such as structured dietary changes and exercise can be effective, but have practical limitations related to long-term adherence. This has underscored the necessity of identifying newer pharmacological candidates and evaluating their comparative efficacy [14,15].

The present study aimed to directly compare the effects of three therapeutic agents - pioglitazone (a selective PPAR-γ agonist), saroglitazar (a dual PPAR-α/γ agonist), and silymarin (a hepatoprotective flavonoid complex) - in a high-fat diet (HFD)-induced rat model of NAFLD, focusing on biochemical, anthropometric, and histopathological endpoints, with special reference to their impact on insulin resistance.

## Materials and methods

Study setting and ethical approval

The experimental study was conducted in January 2024, over an 11-week period after obtaining ethical approval from the Institutional Animal Ethics Committee - Approval no. 184/IAEC/2023, dated 10 October 2023, duration of approved project being 12 months. The study was carried out in the Department of Pharmacology and Therapeutics in collaboration with the Department of Pathology, King George’s Medical University. All animal handling and procedures were performed in accordance with Committee for Control and Supervision of Experiments on Animals (CCSEA) guidelines.

Experimental animals

30 healthy adult male Wistar rats (8-12 weeks old; 150-200 g body weight (BW)) were procured from a CCSEA-registered breeding facility (Indian Institute of Toxicology Research, Lucknow). Animals were acclimatized for one week and housed in polypropylene cages under controlled environmental conditions: 24-27°C temperature, 60-65% relative humidity, and a 12 h light/dark rhythm. Standard pellet diet and water were made available ad libitum. Following acclimatisation, rats were randomised into five equal groups (n = 6/group).

Study groups

**Figure 1:**
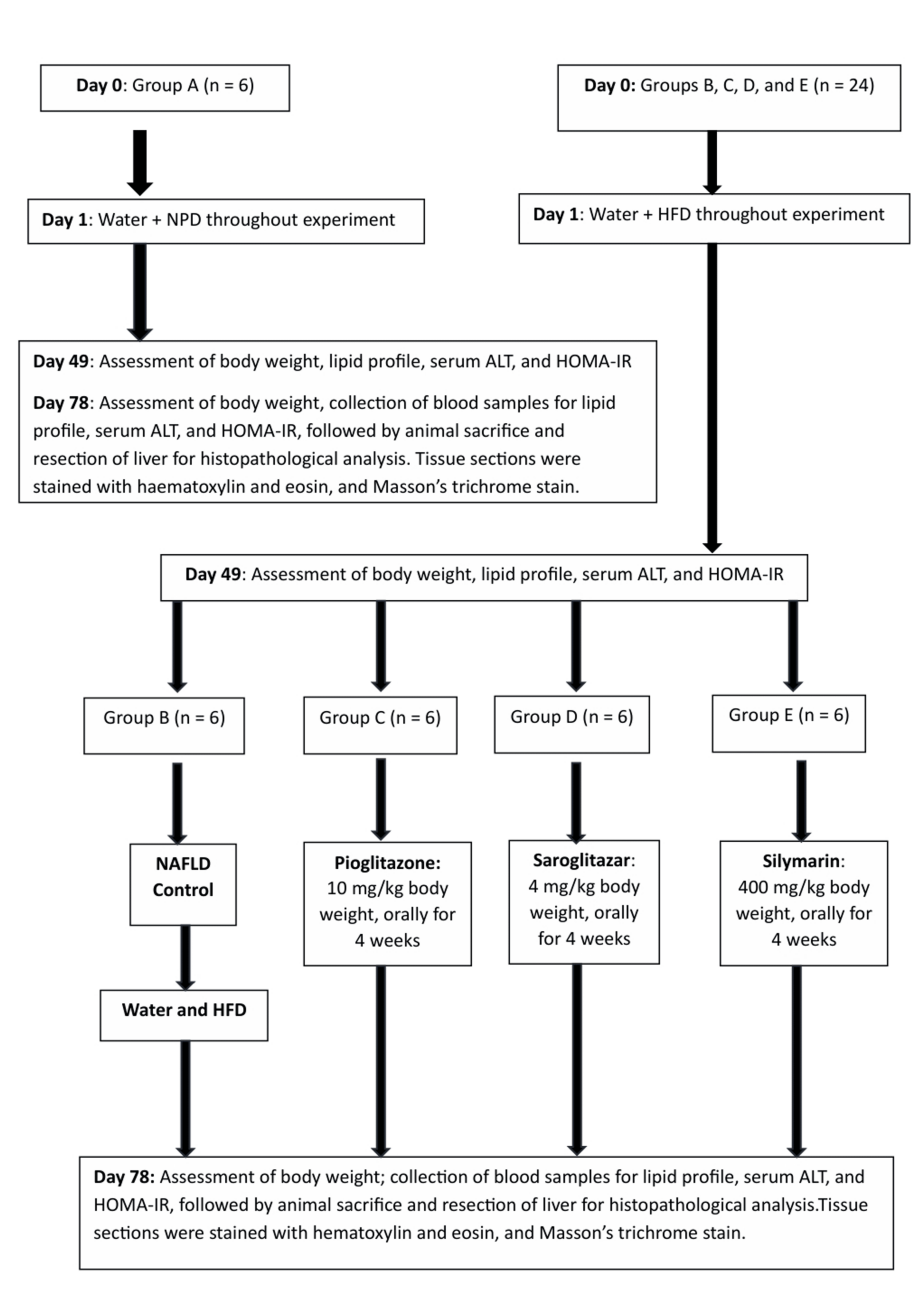
Experimental protocol showing research methodology

Rats were randomly divided into five groups as outlined in Figure 1. Each group containing n=6 rats were randomly allocated as follows: Group A (Normal Control Group) - Rats were fed with NPD and water ad libitum, throughout the study; Group B (NAFLD Control Group) - NAFLD Rats were fed with HFD and water ad libitum, throughout the study; Group C (Pioglitazone Treated Group) - NAFLD Rats were given Pioglitazone with HFD and water ad libitum for 28 days; Group D (Saroglitazar Treated Group) - NAFLD rats were given saroglitazar with HFD and water ad libitum for 28 days; and Group E (Silymarin Treated Group) - NAFLD rats were given silymarin with HFD and water ad libitum.

Induction of NAFLD

Group A (control) received normal pellet diet. The remaining animals (Groups B-E) were maintained on an HFD (58% fat, 25% protein, 17% carbohydrate) with ad libitum water supply for seven weeks to induce NAFLD [16]. NAFLD model validation was performed by biochemical confirmation through lipid profiling at the end of induction.

Drugs and chemicals

The test drugs utilized in this study included pioglitazone, saroglitazar, and silymarin, each prepared according to established protocols. After disease induction, pioglitazone, a thiazolidinedione and selective PPAR-γ agonist, was administered to Group C orally at a dose 10 mg/kg orally dissolved in normal saline and sourced from Cipla Ltd. Saroglitazar, a dual PPAR-α/γ agonist, was also given orally to Group D at a dose of 4 mg/kg BW, prepared in normal saline and supplied by Zydus Healthcare Ltd [17,18]. Silymarin, a standardized flavonolignan extract from *Silybum marianum*, was administered orally to Group E at a dose of 400 mg/kg BW dissolved in 0.2% carboxymethylcellulose, with the compound obtained from Sigma Aldrich Chemicals Pvt. Ltd [19].

All drugs and their respective vehicles were freshly prepared prior to administration and delivered via oral gavage once daily for 28 days following the induction of NAFLD. The control and NAFLD groups received either normal saline and 0.2% carboxymethylcellulose as appropriate vehicle controls. At the end of the intervention period, animals were humanely euthanised by decapitation.

Parameters evaluated

Anthropometric parameters such as BW were measured using a digital weighing balance at designated intervals (Week 7, and Week 11), while liver weight (LW) was determined immediately after euthanasia, and the LW to BW ratio (%) was calculated using the standard formula:

(LW/BW) × 100

Biochemical assessments involved collecting blood samples via established techniques, including retro-orbital plexus sampling and cardiac puncture at Week 7 and Week 11, followed by serum separation and analysis on a semi-automated analyzer. Serum lipid parameters, comprising total cholesterol (TC), triglycerides (TG), and high-density lipoprotein cholesterol (HDL-c), as well as alanine aminotransferase (ALT), were measured using enzyme-based colorimetric end-point kits. Low-density lipoprotein cholesterol (LDL-c) and very low-density lipoprotein cholesterol (VLDL-c) were calculated with the Friedewald formula:

LDL-c = TC - (HDL-c + VLDL-c), and

VLDL-c = TG/5

Fasting blood glucose was assessed by glucometer, serum insulin by chemiluminescent microparticle immunoassay, and insulin resistance was quantified using the Homeostasis Model Assessment of Insulin Resistance (HOMA-IR) formula [20]: 

HOMA-IR = (Fasting Glucose (mg/dL) × Insulin (μU/mL))/405 

Histopathology

Liver samples were preserved in formalin, paraffin embedded, and sectioned for hematoxylin and eosin (H&E) and Masson’s trichrome staining. Steatosis, portal inflammation, and fibrosis were graded using the Dixon and Jahn criterias [17,21,22]. Pathologist remained blinded to treatment allocations.

Statistical analysis

Statistical analysis was performed using GraphPad Prism software (version 8), with all data expressed as mean ± standard deviation. Intergroup differences for continuous variables across the five experimental groups were assessed using one-way analysis of variance (ANOVA) to determine overall statistical significance. Where ANOVA indicated significant differences (p < 0.05), post hoc Tukey’s Honestly Significant Difference (HSD) tests were applied to show specific group comparisons exhibiting statistically significant differences The predefined p-value was set less than 0.05 for statistical significance.

## Results

Anthropometric outcomes

At Week 7, animals on HFD showed a significant rise in mean BW compared to controls (F = 62.65, p < 0.0001). By Week 11, this difference persisted (F = 44.05, p < 0.0001). LW at the end of the study also differed significantly across groups (F = 40.34, p < 0.0001). When corrected for BW, the liver index demonstrated moderate variability (F = 4.11, p = 0.0108) as seen in Table 1. Tukey’s post hoc tests revealed that rats receiving pioglitazone or saroglitazar gained significantly less weight than the silymarin group (p = 0.0091 and p = 0.0044, respectively). Both pioglitazone and saroglitazar groups displayed lower absolute LW compared with untreated NAFLD animals, and saroglitazar significantly reduced LW further compared with silymarin (p = 0.0335).

**Table 1:** Anthropometric parameters of rats in different groups The predefined p-value was set less than 0.05 for statistical significance. BW: Body weight; LW: Liver weight; NAFLD: Non-alcoholic fatty liver disease; ANOVA: Analysis of variance

Experimental Groups	BW (grams)		LW (grams)	LW:BW
	Week 7 (Mean ± SD)	Week 11 (Mean ± SD)	Week 11 (Mean ± SD)	Week 11 (Mean ± SD)
Group A - Control	224.2 ± 10.26	224.7 ± 10.39	6.35 ± 0.31	2.78 ± 0.19
Group B - NAFLD	308.5 ± 11.64	314.5 ± 10.25	8.38 ± 0.34	2.62 ± 0.19
Group C - Pioglitazone	305.2 ± 13.04	273.7 ± 18.17	7.47 ± 0.31	2.77 ± 0.20
Group D - Saroglitazar	312.0 ± 12.18	271.5 ± 9.65	7.07 ± 0.22	2.55 ± 0.10
Group E - Silymarin	313.0 ± 12.05	300.7 ± 13.11	7.58 ± 0.24	2.45 ± 0.15
ANOVA	F = 62.65, p-value <0.0001	F = 44.05, p-value <0.0001	F = 40.34, p-value <0.0001	F = 4.110, p-value = 0.0108

Serum lipids and ALT

**Figure 2:**
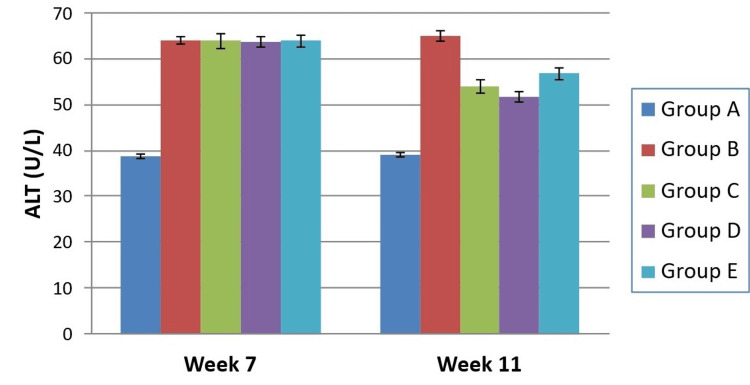
Intergroup comparison of serum ALT at different time intervals ALT: Alanine aminotransferase

At induction (Week 7), HFD groups exhibited marked dyslipidaemia: elevated TC, TG, LDL‑c, and VLDL‑c, along with reduced HDL‑c and raised ALT (all analysis of variance (ANOVA) p < 0.0001). After four weeks of therapy, untreated NAFLD controls maintained the worst lipid profile, whereas all three therapies showed clear biochemical improvement. Intergroup analyses at Week 11 confirmed strong differences for all lipid fractions and ALT (p values < 0.0001). Saroglitazar produced the greatest fall in TC compared with pioglitazone (p = 0.0235) and silymarin (p < 0.0001). For TG, saroglitazar was again superior to the other two drugs (p < 0.001). All active treatments improved HDL‑c significantly over controls, though no inter‑drug differences were detected. LDL‑c reductions were most marked with saroglitazar compared with pioglitazone (p = 0.001). VLDL‑c was also diminished to a greater extent by saroglitazar than pioglitazone (p = 0.001) or silymarin (p < 0.0001) as seen in Table 2. Mean ALT values fell significantly for all interventions; the saroglitazar arm achieved the lowest levels, with differences over both comparators reaching significance as depicted in Figure 2.

**Table 2:** Biochemical parameters of rats in different groups The predefined p-value was set less than 0.05 for statistical significance. TC: Total cholesterol; NAFLD: Non-alcoholic fatty liver disease; ANOVA: Analysis of variance; TG: Triglycerides; HDL-C: High-density lipoprotein cholesterol; LDL-C: Low-density lipoprotein cholesterol; VLDL-C: Very low-density lipoprotein cholesterol; ALT: Alanine aminotransferase

Parameter	Group	Week 7	Week 11
TC (mg/dL)	Group A - Control	36.87 ± 0.87	36.97 ± 0.64
	Group B - NAFLD	53.92 ± 2.64	58.80 ± 1.77
	Group C - Pioglitazone	55.00 ± 1.97	49.28 ± 1.69
	Group D - Saroglitazar	54.89 ± 2.49	46.06 ± 0.98
	Group E - Silymarin	54.83 ± 2.33	52.48 ± 2.66
ANOVA		F = 81.99, p-value <0.0001	F = 135.6, p-value <0.0001
TG (mg/dL)	Group A - Control	40.00 ± 0.45	40.07 ± 0.54
	Group B - NAFLD	60.21 ± 1.52	60.95 ± 1.41
	Group C - Pioglitazone	60.01 ± 2.33	52.94 ± 0.84
	Group D - Saroglitazar	59.28 ± 1.59	48.68 ± 2.73
	Group E - Silymarin	59.48 ± 2.02	56.86 ± 1.65
ANOVA		F = 161.1, p value <0.0001	F = 147.2, p value <0.0001
HDL-C (mg/dL)	Group A - Control	12.82 ± 0.37	12.88 ± 0.38
	Group B - NAFLD	11.18 ± 0.29	11.12 ± 0.24
	Group C - Pioglitazone	11.18 ± 0.27	12.73 ± 0.31
	Group D - Saroglitazar	11.16 ± 0.30	12.76 ± 0.35
	Group E - Silymarin	11.10 ± 0.20	12.50 ± 0.30
ANOVA		F =39.43, p value <0.0001	F = 31.11, p value <0.0001
LDL-C (mg/dL)	Group A - Control	16.04 ± 0.88	16.08 ± 0.70
	Group B - NAFLD	23.04 ± 2.82	27.34 ± 2.09
	Group C - Pioglitazone	24.25 ± 2.67	23.23 ± 1.96
	Group D - Saroglitazar	24.38 ± 2.66	22.13 ± 1.58
	Group E - Silymarin	24.29 ± 2.56	24.28 ± 2.89
ANOVA		F = 13.15, p value <0.0001	F = 26.31, p value <0.0001
VLDL-C (mg/dL)	Group A - Control	8.00 ± 0.09	8.01 ± 0.11
	Group B - NAFLD	12.04 ± 0.30	12.19 ± 0.28
	Group C - Pioglitazone	12.00 ± 0.47	10.59 ± 0.17
	Group D - Saroglitazar	11.86 ± 0.32	9.74 ± 0.55
	Group E - Silymarin	11.90 ± 0.40	11.37 ± 0.33
ANOVA		F = 161.1, p value <0.0001	F = 147.2, p value <0.0001
ALT (IU/L)	Group A - Control	38.72 ± 0.50	39.15 ± 0.45
	Group B - NAFLD	64.07 ± 0.80	64.98 ± 1.03
	Group C - Pioglitazone	63.89 ± 1.55	53.92 ± 1.45
	Group D - Saroglitazar	63.80 ± 1.13	51.71 ± 1.24
	Group E - Silymarin	63.87 ± 1.18	56.73 ± 1.24
ANOVA		F = 639.9, p value <0.0001	F =408.8, p value <0.0001

Insulin resistance

**Figure 3:**
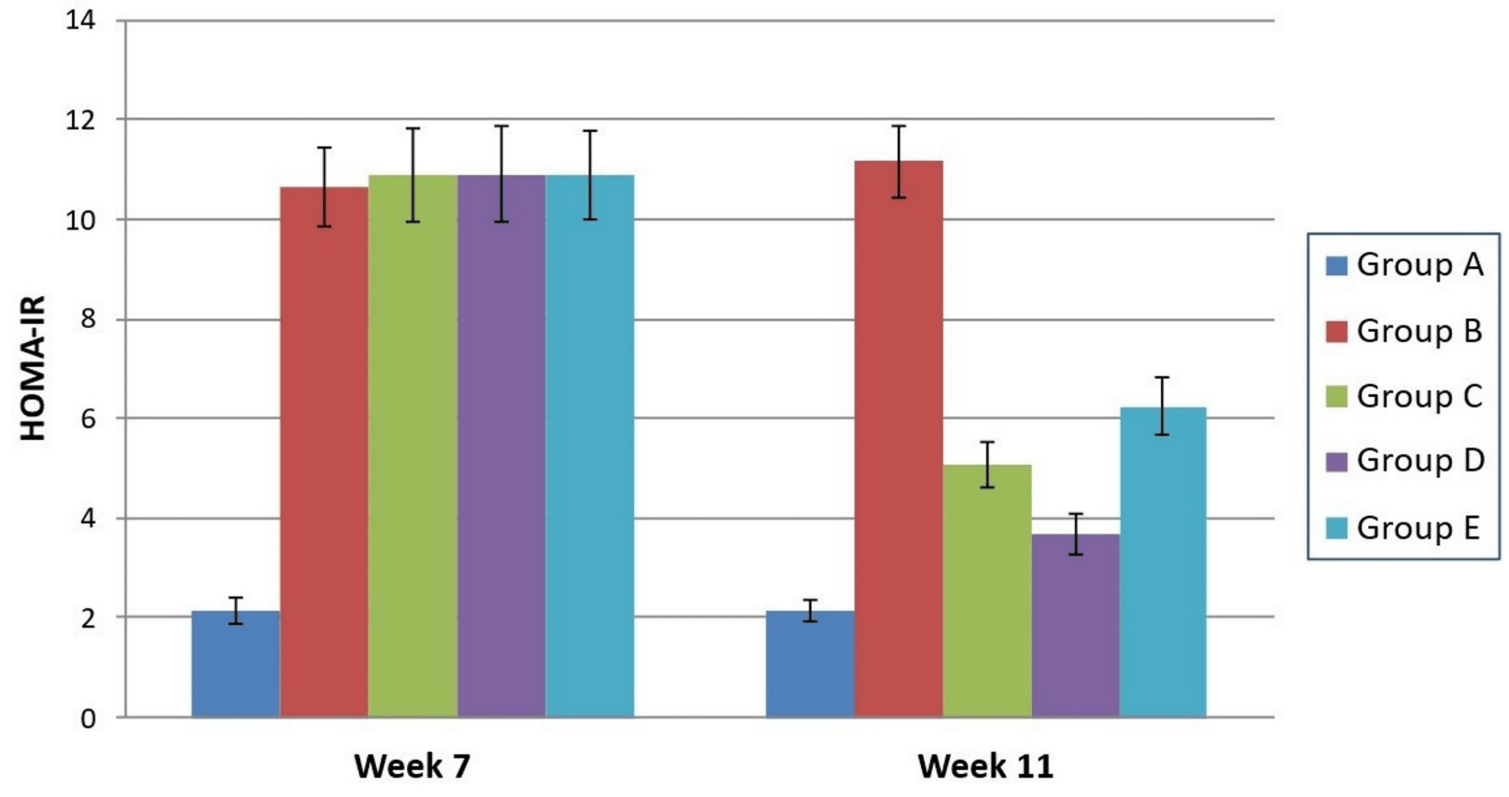
Intergroup comparison of HOMA-IR at different time intervals HOMA-IR: Homeostasis Model Assessment of Insulin Resistance

HOMA‑IR increased significantly by Week 7 in high‑fat‑diet animals relative to controls (F = 135.5, p < 0.0001). Treatment for four weeks led to a decrease in insulin resistance across all therapeutic groups (F = 275.9, p < 0.0001). Saroglitazar showed the maximum impact, reducing HOMA‑IR significantly more than pioglitazone (p = 0.0005) and silymarin (p < 0.0001). Pioglitazone also reduced insulin resistance more effectively than silymarin (p = 0.0048) as seen in Figure 3.

Histopathology

**Figure 4:**
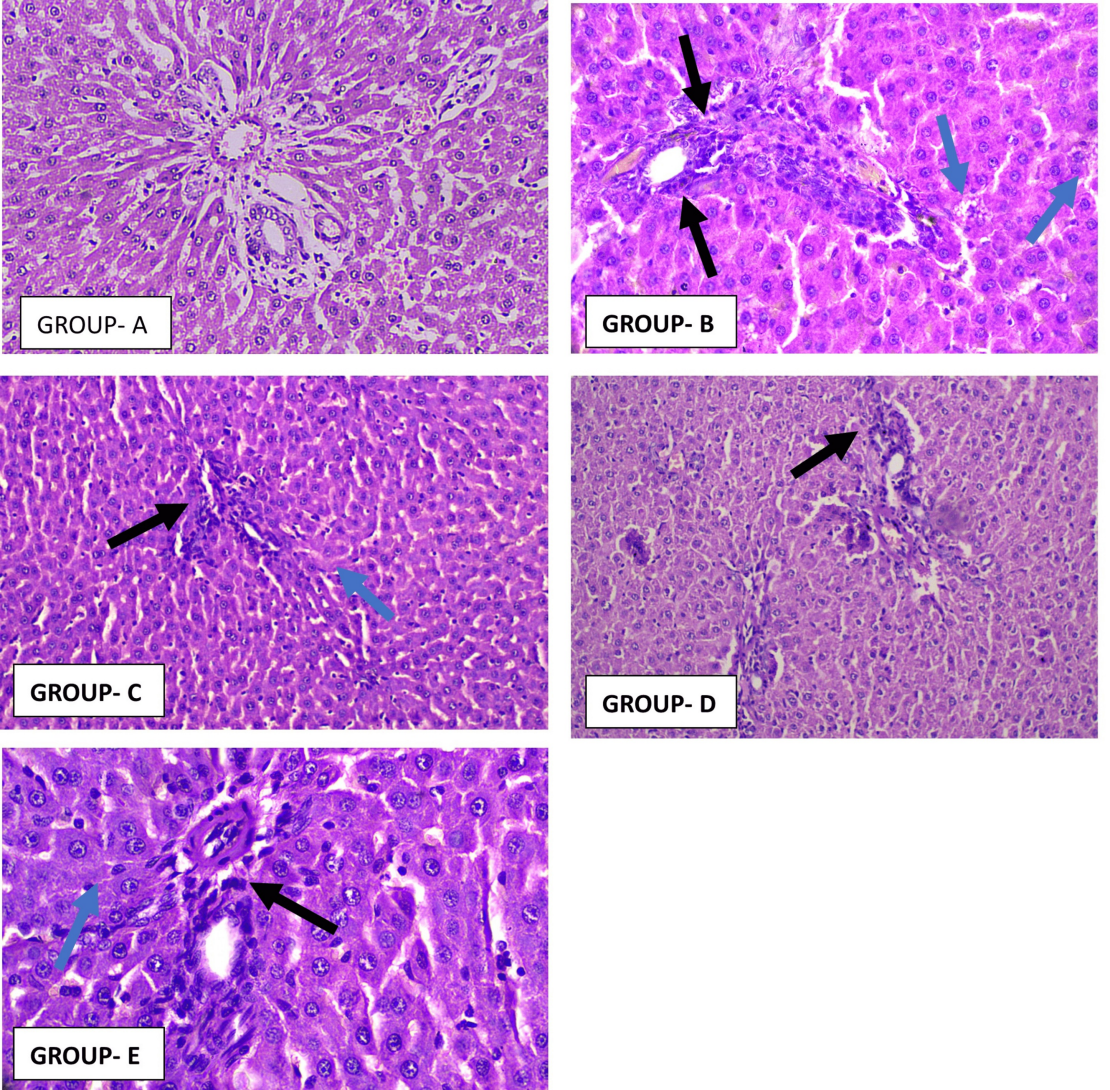
Representative photomicrographs of rat liver section stained with H&E (×40) from Group A To Group E Group A - Liver architecture is preserved with radiating cords of hepatocytes, clear sinusoids, and intact portal tract. There is absence of steatosis, ballooning, or inflammatory infiltrates. Group B - Macrovesicular steatosis is prominent in hepatocytes, with ballooning (blue arrow) and loss of regular architecture. The portal tract shows marked lymphocytic infiltration (black arrow), consistent with steatohepatitis. Group C - Liver tissues display a pronounced decrease in macrovesicular fat deposition and hepatocyte ballooning compared to the NAFLD control. A slight portal lymphocytic presence is noted, and the hepatic cell plates exhibit improved structural organization, suggesting partial tissue recovery. Group D - Histological sections show a significant reduction in large fat droplets and cellular ballooning relative to the NAFLD control. Minimal lymphocytic infiltration is evident around the portal areas, while hepatocyte arrangement appears more uniform, indicating beginnings of histological regeneration. Group E - Sections present a distinct decline in macrovesicular steatosis and hepatocellular ballooning compared with the NAFLD control. Mild portal inflammatory activity persists, yet hepatocytes reveal better alignment of cell cords, denoting partial restoration of liver architecture. H&E: Hematoxylin and eosin; NAFLD: Non-alcoholic fatty liver disease

**Figure 5:**
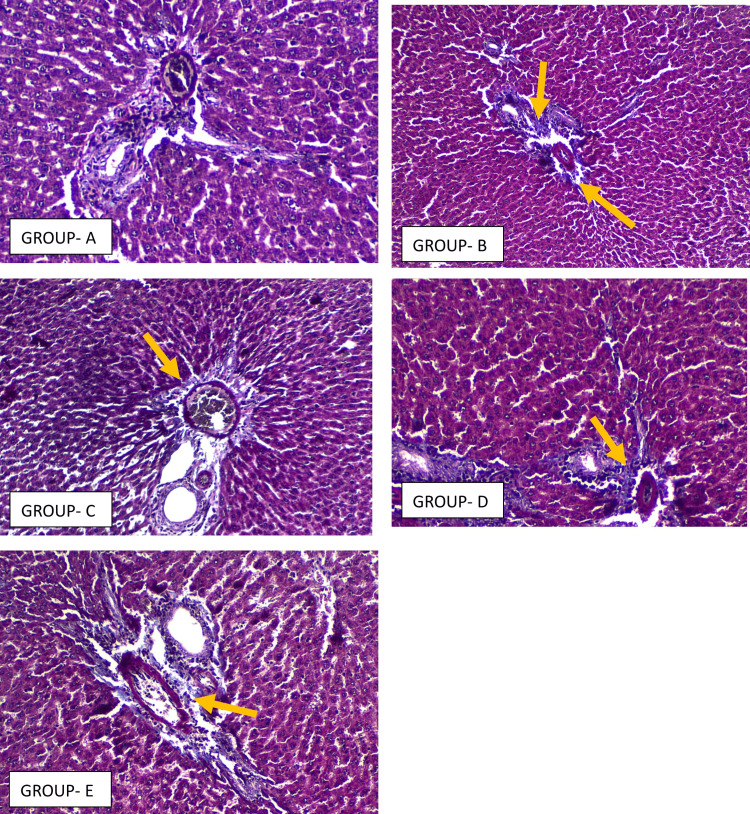
Representative photomicrographs of rat liver section stained with Masson’s trichrome stain (×40) in Group A to Group E Group A - Minimal collagen deposition is confined to the portal tract, consistent with normal tissue; with no perisinusoidal and periportal fibrosis. Group B - Shows extensive periportal and perisinusoidal collagen deposition (yellow arrow), and portal tract expansion, indicating the development of early hepatic fibrosis. Group C - Sections reveal mild to moderate deposition of collagen in the portal and periportal regions. The fibrotic activity appears reduced relative to the untreated NAFLD control, though attenuation is not pronounced. Group D - Liver tissue exhibits limited portal and periportal collagen accumulation, indicating a moderate fibrotic response. Fibrosis remains lesser than in the NAFLD control but not markedly regressed. Group E - Histology shows mild-to-moderate collagen deposition around portal and periportal zones. While the fibrotic reaction is diminished when compared to the untreated NAFLD group, the reduction is only partial. NAFLD: Non-alcoholic fatty liver disease

Liver histology demonstrated extensive changes in the untreated NAFLD cohort. Steatosis, portal inflammation, and fibrosis scores were significantly higher across groups (F values = 671.0, 58.92, 120.1, and 158.1, all p < 0.0001). Saroglitazar led to the most pronounced attenuation of steatosis, with improvements greater than both pioglitazone and silymarin (p < 0.0001). Portal inflammation extent improved with pioglitazone and saroglitazar in comparison with controls, though differences between the two were not significant. For inflammation intensity, saroglitazar and pioglitazone were both superior to silymarin as seen in Figure 4. Fibrosis scores improved across all therapies compared with NAFLD controls as seen in Figure 5; however, inter‑drug differences in fibrosis regression were not statistically significant as seen by the values described in Table 3.

**Table 3:** Histopathological parameters of rats in different groups The predefined p-value was set less than 0.05 for statistical significance. NAFLD: Non-alcoholic fatty liver disease; ANOVA: Analysis of variance

Experimental Groups	Steatosis	Portal Inflammation Extent	Portal Inflammation Intensity	Fibrosis
	Week 7 (Mean ± SD)	Week 11 (Mean ± SD)	Week 11 (Mean ± SD)	Week 11 (Mean ± SD)
Group A - Control	0.13 ± 0.10	0.15 ± 0.05	0.12 ± 0.03	0.10 ± 0.03
Group B - NAFLD	3.71 ± 0.17	1.55 ± 0.32	1.80 ± 0.26	2.10 ± 0.26
Group C - Pioglitazone	3.10 ± 0.15	1.22 ± 0.09	1.35 ± 0.17	2.03 ± 0.18
Group D - Saroglitazar	0.43 ± 0.09	1.10 ± 0.08	1.10 ± 0.08	1.95 ± 0.11
Group E - Silymarin	2.95 ± 0.24	1.30 ± 0.17	1.66 ± 0.09	2.06 ± 0.18
ANOVA	F = 671.0, p-value <0.0001	F = 58.92, p-value <0.0001	F = 120.1, p-value <0.0001	F = 158.1, p-value <0.0001

## Discussion

This study demonstrates that all three agents - pioglitazone, saroglitazar, and silymarin - provided therapeutic benefit in an HFD rat model of NAFLD. Compared to untreated NAFLD controls, pioglitazone and saroglitazar were more effective in reversing pathological weight gain and reducing liver enlargement, with saroglitazar showing a marginally superior impact on metabolic regulation and histological recovery. All treated groups exhibited improvements in serum lipid profiles, reductions in liver enzyme levels, and enhanced insulin sensitivity, indicating amelioration of underlying metabolic dysfunction. Histologically, saroglitazar and, to a lesser extent, silymarin achieved pronounced reduction in hepatic steatosis, inflammation, and fibrosis, highlighting the additive benefit of dual-action and antioxidant approaches.

Previous experimental evidence has demonstrated that saroglitazar significantly improves hepatic steatosis and fibrosis by modulating inflammatory cytokines and adiponectin pathways in animal models of non-alcoholic steatohepatitis [23]. The study results correspond well with the work of Collino et al., who demonstrated that pioglitazone improved lipid profiles by decreasing LDL-c and TG while increasing HDL-c, and reduced hepatic inflammation and insulin resistance via PPAR-γ activation in experimental rats [24]. These findings align with the present study, where pioglitazone significantly reduced serum lipid parameters and ALT levels.

Further support comes from diet-induced NAFLD models in which saroglitazar improved insulin resistance and attenuated steatohepatitis through dual PPAR-α/γ agonism [25].

With regard to silymarin, prior meta-analyses have shown that silymarin significantly reduces transaminase levels and improves lipid profiles in NAFLD patients [13]. Experimental studies using advanced formulations, including silymarin-loaded chitosan nanoparticles, have further demonstrated hepatoprotective effects mediated through antioxidant and anti-inflammatory mechanisms [26]. These observations are consistent with the present study, where silymarin improved biochemical parameters and hepatic steatosis, though its effects on advanced fibrosis were modest.

In this short‑duration, HFD-induced NAFLD rat model, no overt toxicity or mortality was observed but the limited time frame and sample size preclude robust conclusions about chronic adverse effects. Longer‑term studies incorporating detailed toxicity endpoints and dose‑response evaluation are therefore needed to more clearly define the safety profiles of these agents in NAFLD.

The strengths of the present study lie in its comprehensive and systematic design, offering a direct comparative assessment of pioglitazone, saroglitazar, and silymarin - three agents with distinct mechanisms of action - using a standardized HFD-induced NAFLD rat model. The inclusion of anthropometric, biochemical, and histopathological endpoints provides strong translational relevance and supports future individualized therapeutic strategies.

Study limitations

The findings are tempered by limitations such as short duration of monotherapy regimens, which constrains conclusions about long-term efficacy, potential drug synergies and a lack of long-term fibrosis outcome data, suggesting the need for continued research to optimize pharmacological strategies in NAFLD.

## Conclusions

While preclinical in scope, the study's results are highly pertinent for informing current and future paradigms in NAFLD therapy, policy formulation, and translational research, ultimately advancing the goal of comprehensive, individualized management of this complex metabolic disorder. The pathophysiological convergence between rat NAFLD models and the human condition, especially regarding insulin resistance, lipid metabolism, oxidative stress, and chronic inflammation, enables cautious extrapolation of these preclinical findings to human populations. From a clinical practice perspective, the study reinforces several actionable points like therapeutic selection, combination therapies and hepatic biochemical hepatic end points. The significance of the study emerges most strongly in its translational potential. By directly comparing three agents with distinct mechanisms - metabolic modulators and antioxidant therapy - in a rigorously induced disease model, this work bridges the gap between mechanistic understanding and real-world applicability. It provides a template for personalized, mechanism-based approaches to NAFLD, stimulating both immediate changes in clinical practice and guiding multidisciplinary, multimodal research agendas aimed at reversing the global trajectory of this disease.
